# Oral clonazepam versus lorazepam in the treatment of methamphetamine-poisoned children: a pilot clinical trial

**DOI:** 10.1186/s12887-020-02441-x

**Published:** 2020-12-03

**Authors:** Fariba Farnaghi, Razieh Rahmani, Hossein Hassanian-Moghaddam, Nasim Zamani, Rebecca McDonald, Narges Gholami, Latif Gachkar

**Affiliations:** 1 Department of Pediatrics, Loghman-Hakim Hospital, School of Medicine, Shahid Beheshti University of Medical Sciences, Tehran, Iran; 2 Social Determinants of Health Research Center, Shahid Beheshti University of Medical Sciences, Tehran, Iran; 3 Department of Clinical Toxicology, Loghman-Hakim Hospital Poison Center, School of Medicine, Shahid Beheshti University of Medical Sciences, South Karegar Street, Kamali St, Tehran, Iran; 4 National Addiction Centre, Institute of Psychiatry, Psychology and Neuroscience, King’s College London, London, UK; 5 Infectious Diseases and Tropical Medicine Research Center, Shahid Beheshti University of Medical Sciences, Tehran, Iran

**Keywords:** Benzodiazepine, Clonazepam, Lorazepam, Treatment, Agitation, Methamphetamine, Toxicity

## Abstract

**Objectives:**

To evaluate the efficacy of oral clonazepam versus oral lorazepam following initial parenteral benzodiazepine administration to control methamphetamine-induced agitation in children.

**Methods:**

In a single-center clinical trial, intravenous diazepam (0.2 mg/Kg) was initially administered to all methamphetamine-poisoned pediatric patients to control their agitation, followed by a single dose of oral clonazepam (0.05 mg/Kg; *n* = 15) or oral lorazepam (0.05 mg/Kg; *n* = 15) to prevent relapse of toxicity.

**Results:**

The median age [IQR] (range) was 15 [10, 36] (6-144) months. The source of poisoning was methamphetamine exposure from oral ingestion in 23 (76.7%) and passive inhalation in 7 (23.3%) patients. The most common symptoms/signs were agitation (29; 96.7%), mydriatic pupils (26; 86.7%), and tachycardia (20; 66.6%). Two in each group (13.3%) needed re-administration of intravenous diazepam due to persistent agitation. There was no report of benzodiazepine complications in either group.

**Conclusions:**

Clonazepam and lorazepam treatment was equally effective at similar doses. However, considering the higher potency of clonazepam, it seems that lorazepam is the safer benzodiazepine for oral maintenance treatment of methamphetamine-induced agitation in children and can be used with minimal complications.

**Trial registration:**

IRCT20180610040036N2, April 18th, 2020. Retrospectively registered.

## Background

In recent years, Iran has seen a rise in the prevalence of stimulant abuse, including from methamphetamine, methylphenidate, and ecstasy [1–3]. The hidden nature of stimulant abuse among family members has also resulted in a dramatic increase in the frequency of accidental stimulant toxicity in children. Even though accidental opioid poisonings remain more common in Iranian children [1], this change in adult drug use patterns presents a challenge for clinical practice, since no appropriate antidote exists for stimulant poisoning [4]. The most common signs and symptoms of stimulant toxicity in children are irritability, agitation, hyperactivity, ataxia, seizure, inconsolable or constant body movements, roving eye movements, cortical blindness, hyperthermia, tachycardia, hypertension, vomiting, respiratory distress, and rhabdomyolysis [4].

Benzodiazepines (BZOs) are the first-line medications in the treatment of toxicity from stimulants, including methamphetamine. Management of agitation is the cornerstone in the treatment of methamphetamine poisoning, which can prevent further complications including hyperthermia, hypertension, hallucination, delirium, and rhabdomyolysis.

BZO treatment can control methamphetamine-induced agitation and prevent seizures simultaneously. The binding of BZOs to the GABA receptor increases chloride permeability causing an influx of chloride ions intracellularly and result in anti-anxiety, anti-convulsive, and sedative effects [5]. They are generally intravenously administered until the patient becomes symptom-free and calm.

However, in pediatric patients, access to and maintenance of the intravenous (IV) line is a major concern, especially in younger children and in busy wards. A child may not cooperate with the treating team, and the IV line may be lost during the treatment process due to the child’s movements. IV administration of BZOs in children needs to be slow and requires respiratory monitoring, as rapid administration of BZOs may induce respiratory depression and apnea [6–9]. This risk is not common with oral BZOs [6].

Early administration of oral BZOs has been advocated in adult patients with methamphetamine poisoning [10]. However, the role of oral BZOs in the treatment stimulant-poisoned children after initial emergency department (ED) management is unclear, as literature on this subject is sparse.

While both BZOs can treat anxiety, the main difference is their duration of action. From a clinical point of view, methamphetamine toxicity can present with different symptoms and signs, and it is unclear which BZO is best suited for the treatment of methamphetamine poisoning. Generally, lorazepam is used for sedation, and clonazepam is used to treat anxiety. Lorazepam binds to the GABA-A receptor with greater affinity than clonazepam [11].

In clinical practice, we have observed that initial IV administration of BZOs does not sedate the child or that it can lead to a recurrence of stimulant toxicity. We hypothesize that the combination of two BZOs may have greater efficacy and safety, in which IV administration acts like a loading dose for oral treatment.

The aim of the current study was thus to evaluate the efficacy of oral BZOs in the treatment of methamphetamine poisoning in children referred to the only pediatric poisoning center in Tehran (Iran) after they were initially managed by administration of IV diazepam. For this purpose, we assigned patients to two oral BZO treatments (clonazepam vs. lorazepam) and compared their efficacy in terms of reducing agitation and other manifestations of stimulant toxicity.

## Methods

Thirty methamphetamine-poisoned children who had been referred to Loghman-Hakim Poison Center in Tehran (Iran) between January 2017 and January 2018 were enrolled in this prospective pilot clinical trial adhering to CONSORT guidelines (Iranian Registry of Clinical Trials ID: IRCT20180610040036N2).

Patients were eligible for inclusion in the study if they were below the age of 12 years and met the diagnostic criteria for methamphetamine poisoning. Diagnosis of methamphetamine poisoning was based on summation of three criteria: the history given by the child’s parents, a positive urine methamphetamine test, and clinical presentation. Patients with mixed poisoning (i.e., methamphetamine and other substance) or those whose urine was negative for methamphetamine were excluded from the study. Normal vital signs were determined based on pre-defined measures for each patient’s respective age group [12].

### BZO treatment

All thirty patients were initially administered IV diazepam (0.2 mg/Kg) and then consecutively (on a weekly basis) assigned to either the oral clonazepam or the oral lorazepam group (1:1 allocation ratio, 15 patients each, 0.05 mg/Kg [routine dose of oral BZO in children]). After the initial IV diazepam treatment, oral BZOs were only given once to prevent return of stimulant toxicity by one of three of co-authors on shift. They were given half an hour after the patient was calm and could be switched to oral regimen. If the child was not sedated sufficiently after 45 minutes, we then administered next dose of IV diazepam.

### Data collection

Records were kept on the patients’ demographic characteristics, urine drug screen results, route of methamphetamine exposure (inhalation versus ingestion), time elapsed between methamphetamine use and hospital presentation, vital signs and signs/symptoms on presentation, type and total dose of the oral BZO administered (clonazepam versus lorazepam), need for re-administration of the IV BZOs after initial management of the patient, time elapsed between BZO administration and resolution of the signs and symptoms, duration of hospital stay, and side effects of treatment (respiratory depression, deep sedation, and paradoxical agitation).

### Patient monitoring

Patients were continuously monitored for pulse rate, respiratory rate, blood pressure and cardiac rhythm. However, since the children were irritable and using pressure cuffs for blood pressure check could agitate them, blood pressure was checked only hourly for the first six hours following admission and then subsequently in six-hour intervals until discharge. During the post-treatment observation period, the nurses would call the attending physician if they detected agitation or any changes in the patient’s vital signs or cardiac rhythm. Patients’ monitoring continued till discharge.

### Data analysis

The data were analyzed using IBM Statistical Package for Social Sciences (SPSS) version 21. For qualitative variables, percentage of frequency was reported. Mann-Whitney U-test and chi-square test were used to evaluate the association between continuous and categorical variables. The Friedman’s two-way ANOVA was used to test for differences between lorazepam and clonazepam to see timeline difference for respiratory rate, heart rate, temperature, and blood pressure. A *P* value of less than 0.05 was considered to be statistically significant.

## Results

A total of 30 patients were enrolled, with 15 patients evaluated in each group. Nineteen (63.4%) were male. Their age ranged from 6 to 144 months (median 15; IQR [10, 36]).

Twenty-three patients (76.7%) had ingested methamphetamine, either in the form of crystal powder (16 patients; 53.3%) or as water from a methamphetamine pipe (7 patients; 23.3%). Seven (23.3%) had been passively exposed to methamphetamine smoked by their parents (i.e., the fathers, based on patient history).

Most exposures had happened at night (25 cases; 83.3%), including all passive smoking cases (7 cases; 23.3%), 14 cases (46.7%) of crystal powder ingestion, and 4 cases (13.3%) of pipe water ingestion.

The median elapsed time [IQR] between exposure and development of methamphetamine toxicity was one hour [1, 2] (30 minutes to 24 hours), and the median [IQR] time between exposure and hospital presentation was five hours [3, 6] (one hour to 72 hours; Table 1), according to parental report. Urine methamphetamine was positive in all cases.

**Table 1:** Demographics, on-arrival presentation, and treatment response by group

Variable	Total (*n* = 30)	Lorazepam (*n* = 15)	Clonazepam (*n* = 15)	*P* (MWU test)
	Median [IQR] (range)			
Age (months)	15 [10, 36] (6, 144)	15 [10, 37] (6, 144)	17 [11, 33] (6, 84)	0.637
Weight (kg)	11 [10,14] (6.5, 40)	11.5 [9.5, 15] (8, 40)	10.7 [10,, 13.7] (6.5, 25)	0.697
Time elapsed between exposure and presentation (h)	3 [5,6] (1, 72)	5 [4,9] (2, 72)	3 [5,6] (1, 14)	0.377
Time elapsed between exposure and the development of symptoms (h)	1 [1,2] (0.5, 24)	1 [1,, 2.2] (0.5, 24)	1 [1,2] (0.5, 2)	0.275
**Presentation on arrival**				
Temperature (C)	37 [36.5, 37] (36, 37.7)	36.9 [36.5, 37] (36, 37.3)	37 [36.7, 37.1] (36.5, 37.5)	0.142
Systolic BP (mmHg)	90 [90, 100] (80, 110)	92 [90, 100] (80, 110)	90 [90, 100] (80, 110)	0.400
Diastolic BP (mmHg)	50 [50, 60] (40, 80)	60 [50, 70] (50, 80)	50 [50, 60] (40, 70)	0.160
Heart rate (per minute)	127 [110, 140] (90, 160)	130 [110, 150] (92, 160)	120 [102, 140] (90, 150)	0.498
Respiratory rate (per minute)	30 [27, 35] (16, 60)	31[26, 35] (22, 60)	30 [28, 34] (16, 60)	0.667
Creatine phosphokinase (U/L)	218 [148, 360] (88, 1584)	225 [148, 461] (117, 698)	211[126, 324] (88, 1584)	0.697
**Treatment response**				
Duration of symptoms (h)	4 [3,5] (2, 20)	3 [2,4] (2, 13)	5 [3,7] (2, 20)	0.166
Hospitalization period (h)	24 [24, 48] (24, 72)	24 [24, 48] (24, 48)	24 [24, 42] (24, 72)	0.525

The most common signs and symptoms of toxicity were agitation (29 patients; 96.7%) followed by mydriatic pupils (26; 86.7%), tachycardia (20; 66.7%), insomnia (18; 60%), stereotypical movements (hand shaking, waving, or wringing, head banging, self-hitting, and self-biting;12; 40%), tachypnea (8; 26.7%), vomiting (7; 23.3%), and talkativeness (5; 16.6%). Other important signs and symptoms were delusion, tremor, and sweating (each in two patients; 6.7%), and hallucinations and seizure (each in one patient; 3.3%). One case (3.3%) had hyperthermia (axillary temperature > 37.5 Ċ) on presentation. On arrival, hypotension was present in one patient (3.3%) and hypertension in another (3.3%). Three patients (9.9%) had low diastolic blood pressures (DBP). Rhabdomyolysis (CPK > 1000 U/L) was reported in one patient (3.3%).

### Treatment response

After initial administration of 0.2 mg/Kg IV diazepam in all patients, four patients (2 in each group) needed re-administration of IV diazepam (at 45 minutes, 50 minutes, 60 minutes, and 75 minutes after the first diazepam dose) due to persistent agitation after the first dose.

Oral BZOs were administered only once immediately after the patients became calm and could be switched to oral regimen (mean 1 hour; range, 0.5 to 3 h). 15 patients received oral clonazepam (0.05 mg/Kg) and another 15 received oral lorazepam (0.05 mg/Kg). The mean administered dose of oral BZO was 1.1 mg in both groups.

Statistical analysis showed that vital signs were similar between the two groups on arrival and after BZO treatment (see Table 1; all *P*s were higher than 0.05).

Almost 73% (22 cases) of patients responded to treatment within five hours of administration of the oral BZOs. All patients remain conscious during observation period and no adverse effects were seen following oral BZOs administration. In three cases (10%), symptoms persisted for 12 hours or more (i.e., up to 20 hours). Although the median duration of symptoms was less in those treated with lorazepam (3 versus 5 hours), the difference was not significant (p = 0.166).

Table 2 shows vital signs (including respiratory rate) during the hospitalization period in 6-hour intervals, following IV diazepam treatment as well as initiation of oral BZO treatment.

**Table 2:** Vital signs over time (6-hour intervals) post benzodiazepine treatment (*n* = 30)

Variable	BZO	Time post initiation of oral benzodiazepine (hours)					*p*-value*	Pairwise comparison
		T0	T6	T12	T18	T24		
Heart rate^a^	Lorazepam	**130 [110, 150]** **(90, 160)**	**112 [110, 128]** **(100, 140)**	110 [102, 112] (90, 120)	**102 [100, 112]** **(88, 130)**	**100 [99, 110]** **(80, 126)**	< 0.001	T0 vs. T24 T6 vs. T24 T0 vs. T18
	Clonazepam	120 [100, 140] (92, 144)	115[106, 122] (100, 148)	107 [100, 110] (80, 127)	110 [97, 119] (88, 123)	108 [98, 120] (88, 120)	0.673	
Temperature^a^	Lorazepam	37 [36.6, 37] (36, 37.1)	**37 [36.9, 37]** **(36.7, 37.8)**	36.8 [36.5, 37] (36.2, 37.5)	36.8 [36.5, 37] (36.2, 37.5)	**36.5 [36.5, 36.8]** **(36.2, 37)**	0.002	T6 vs. T24
	Clonazepam	37 [36.6, 37] (36, 37.2)	37 [36.9, 37] (36.5, 37)	36.8 [36.5, 37] (36, 37.2)	37 [36.5, 37] (36.5, 37.2)	36.5 [36.5, 36.9] (36.5, 37)	0.242	-
Respiratory rate^a^	Lorazepam	**30 [26, 35]** **(20, 60)**	25 [22, 27] (20 ,55)	**25 [22, 25]** **(18 ,50)**	23 [22, 26] (20 ,26)	**22 [22, 24]** **(21 ,25)**	0.006	T0 vs. T12 T6 vs. T24
	Clonazepam	**30 [24, 33]** **(16, 60)**	**29 [20, 32]** **(20 ,40)**	25 [21, 29] (18 ,35)	25 [21, 29] (18 ,30)	**24 [19, 25]** **(18 ,30)**	0.003	T0 vs. T24 T6 vs. T24
Systolic blood pressure^a^	Lorazepam	92 [90, 100] (80, 100)	92 [90, 90] (80, 100)	90 [80, 97] (80, 100)	90 [75, 100] (70, 100)	90 [77, 92] (70, 100)	0.647	-
	Clonazepam	90 [90, 100] (80, 110)	90 [90, 100] (80, 110)	85 [80, 90] (80, 100)	85 [80, 85] (70, 90)	75 [70, 75] (70, 100)	0.160	-

^*^Using Friedman’s two-way ANOVA

^a^Median [IQR] (min, max)

**Fig. 1:**
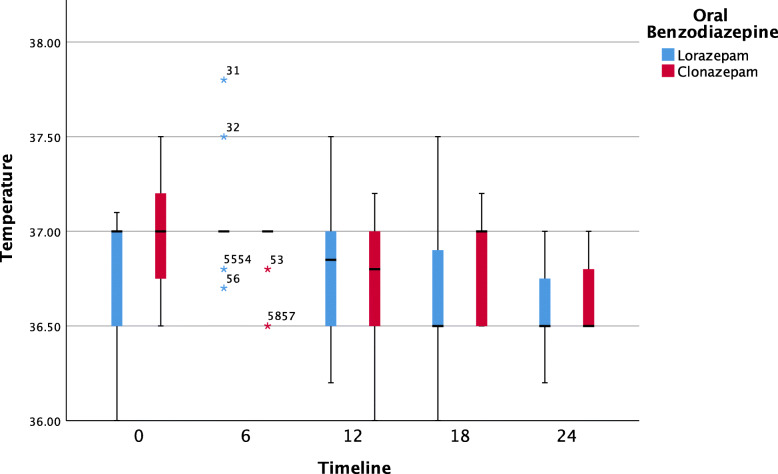
Body temperature over time (6-hour intervals) post benzodiazepine treatment (*n* = 30)

**Fig. 2:**
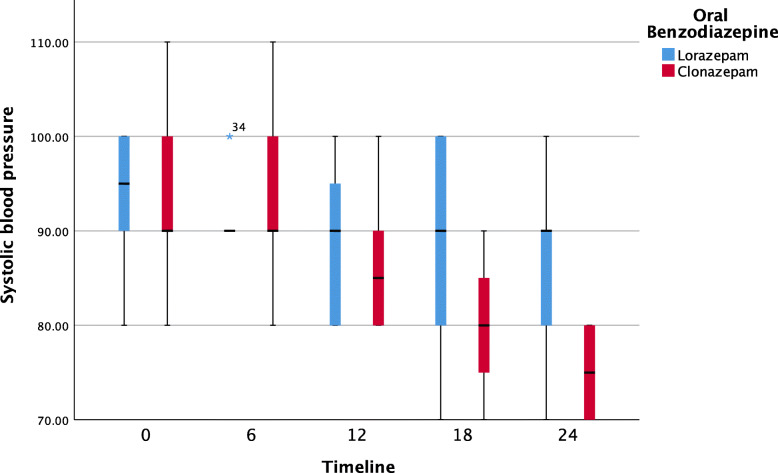
Systolic blood pressure over time (6-hour intervals) post benzodiazepine treatment (*n* = 30)

**Fig. 3:**
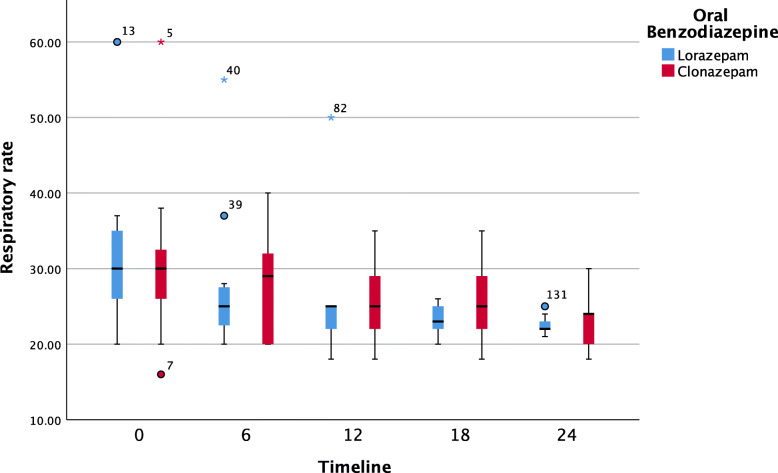
Respiratory rate over time (6-hour intervals) post benzodiazepine treatment (*n* = 30)

**Fig. 4:**
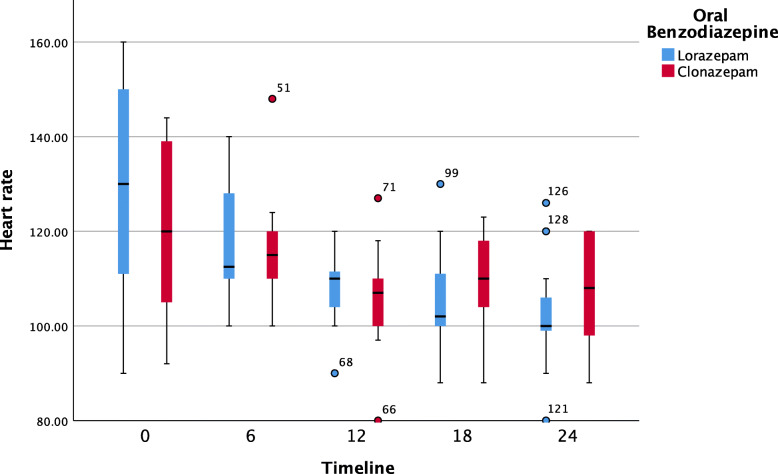
Heart rate over time (6-hour intervals) post benzodiazepine treatment (*n* = 30)

Further pairwise analyses showed that neither oral BZOs had a significant impact on systolic blood pressure over the time. Both clonazepam and lorazepam were effective at decreasing the respiratory rate. The median [IQR] (range) hospitalization period was 24 [24, 48] (24, 72) hours, with 10 patients (33.3%) remaining hospitalized for 24–72 hours. The duration of the hospital stay did not differ significantly between the groups (median: 24 hours, p = 0.525; see Table 1). Figures 1, 2, 3 and 4 show pairwise multiple comparison of the vital signs for both groups in 6-hour time intervals.

## Discussion

In our study, treatment with oral clonazepam and oral lorazepam had the same efficacy in the resolution of the signs and symptoms of methamphetamine toxicity in children. Both treatments could reduce respiratory rate with no significant effect on blood pressure, thus constituting what appears to be appropriate control of agitation. Lorazepam was superior to clonazepam in decreasing patients’ heart rate and temperature. Limited access to parenteral lorazepam (as a good substitute for parenteral BZOs such as diazepam and midazolam) in Iran had had made us look for safer oral alternatives, and our results show that most of our pediatric patients were sedated adequately with the combination treatment of only 0.2-mg/Kg IV diazepam as loading dose followed by oral BZO administration (clonazepam or lorazepam at 0.05 mg/Kg). This combination treatment was well tolerated, as none of the patients in our sample experienced any complications. Oral lorazepam and clonazepam were both administered at an average dose of 1.1 mg. Since we found equal efficacy with both agents at equal dose, information from the BZO equivalency table suggests that lorazepam, as the oral BZO with a wider safety margin, should be preferred [12].

To the best of our knowledge, this pilot study is the first to investigate the efficacy of oral BZOs in pediatric methamphetamine poisoning.

Literature on the management of stimulant-poisoned children has been limited to reports of IV BZO treatment to date. For instance, Van Rijwijk (10-mg diazepam), Duffy MR (5-mg diazepam and 2-mg IV lorazepam), Campbell (Diazepam and IV lorazepam), Cooper (IV diazepam), and Bedford Russel (2.5 mg/kg IV diazepam) have previously evaluated the effectiveness of IV BZO treatment in ecstasy-poisoned children [13–17]. Kung and colleagues reported on a 5-year-old MDMA-poisoned patient with hypertension, tachycardia, hyperthermia, and mydriasis. An initial IV dose of 2.5 mg of diazepam was administered followed by other divided doses in 20-minute intervals which recovered the patient. The patient was discharged home after four days of ICU admission completely symptom-free [18]. Strommen and colleagues reported a 17-month-old infant with methamphetamine poisoning who referred with acute irritability, muscle twitching, and severe perspiration whose agitation was controlled within 30 to 40 minutes after administration of parenteral BZOs [19]. Matteucci et al. retrospectively evaluated 47 pediatric cases (age 0–6 years) of methamphetamine poisoning who had been referred to poisoning control centers in the USA between 2004 and 07, with agitation as most common symptom. Parenteral BZO had been administered to more than half of them, and the mean time to resolution of their signs and symptoms was 22 hours. No death was reported [20]. Ruha and Yarema reported 18 children poisoned with methamphetamine who were admitted to a critical care unit between 1997 and 2004. They were all treated with BZOs, and haloperidol was also administered to 12. They all improved with no important side effect [21].

As depicted in Table 1, the median [IQR] treatment response and duration of symptoms was 4 [3, 5] hours for all patients with a hospitalization period closer to 24–48 hours. The long hospitalization period compared to the short duration of toxicity was due to the observation period to monitor for possible adverse effects (i.e., over-sedation) and persisting agitation. During the monitoring period, patients were mostly calm, but their vital signs (i.e., temperature and heart rate) changed over time, see Table 2. Our findings thus support our hypothesis that treatment with oral clonazepam or lorazepam following an IV diazepam loading dose is efficacious. It may also reduce the burden for healthcare workers, for whom establishing and maintaining the IV line in pediatric patients is a major concern.

Compared to the aforementioned studies, we managed to enroll a substantial sample of 30 patients. Replication of our findings would lend further support to the concept of the combination treatment of IV and oral BZO as a practical, novel, and cost-efficient intervention. However, further studies to evaluate different BZOs in the setting of acute methamphetamine poisoning in children are also warranted. These may include a comparison of the non-IV administration of different BZOs, e.g., by the intranasal (diazepam, midazolam), buccal and intramuscular (both midazolam) routes, which have already been tested in the management of epileptic seizures in children and shown similar efficacy as IV diazepam for this indication [22].

The generalizability of our findings is limited by the fact that all patients were recruited at a single center in Tehran, and the external validity may be questionable. Due to the wide age distribution and limited size of our sample we were unable to stratify outcomes by age range.

Moreover, individual patients were not randomly assigned to their treatment condition (i.e., randomization was conducted based on week of presentation), and we thus cannot rule out the possibility of clinician bias. Future research could compare the treatment regimens more systematically in a blinded RCT. Lack of administration equivalent doses of clonazepam and lorazepam was also a possible limitation. We followed textbook recommendation for recommended dose (mg/kg) of lorazepam and clonazepam, in which they were the same.

Another limitation is lack of agitation score in different times study which should be mentioned in future studies.

## Conclusions

This study demonstrates that oral lorazepam and clonazepam are effective and safe adjunct medications to IV diazepam in the treatment of methamphetamine poisoning and its agitation syndrome in children. Oral BZOs are effective for controlling methamphetamine-induced agitation in children and have no major side effects.

## Data Availability

The datasets analysed during the current study is available from the corresponding author on reasonable request. Full trial protocol can be accessed on https://www.irct.ir/search/result?query=IRCT20180610040036N2.

## Abbreviations

BZO: Benzodiazepine
CPK: Creatine Phosphokinase
DBP: Diastolic Blood Pressure
ED: Emergency Department
GABA: Gamma Aminobutyric Acid
IV: Intravenous
IQR: Inter Quartile Range
MDMA: Methylene Dioxy-Methamphetamine
RCT: Randomized Clinical Trial
SBP: Systolic Blood Pressure
SPSS: Statistical Package for Social Sciences
